# Molecular and Functional Characterization of Pyrokinin-Like Peptides in the Western Tarnished Plant Bug *Lygus hesperus* (Hemiptera: Miridae)

**DOI:** 10.3390/insects12100914

**Published:** 2021-10-06

**Authors:** J. Joe Hull, Colin S. Brent, Man-Yeon Choi, Zsanett Mikó, József Fodor, Adrien Fónagy

**Affiliations:** 1 Pest Management and Biocontrol Research Unit, USDA-ARS, Maricopa, AZ 85138, USA; colin.brent@usda.gov; 2 Horticultural Crops Research Unit, USDA-ARS, Corvallis, OR 97331, USA; man-yeon.choi@usda.gov; 3 Plant Protection Institute, Centre for Agricultural Research, Eötvös Loránd Research Network (Formerly Affiliated with the Hungarian Academy of Sciences), 1051 Budapest, Hungary; miko.zsanett@atk.hu (Z.M.); fodor.jozsef@atk.hu (J.F.); fonagy.adrien@nagyzl.hu (A.F.)

**Keywords:** pyrokinin, FXPRLamide, PK2, *Lygus hesperus*, plant bug, Hemiptera, receptor activation, pheromonotropic activity

## Abstract

**Simple Summary:**

Neuropeptides regulate most insect biological functions. One such group of peptides, the pyrokinins (PKs), are distinguished by a C-terminal FXPRLamide. While widely distributed in most insects, they are poorly characterized in plant bugs. To address this limitation, we identified the PK transcript in the western tarnished plant bug (*Lygus hesperus*) and examined its expression. The *Lygus* PK transcript is predicted to yield three PK-like peptides but only two (LyghePKa and LyghePKb) have the characteristic C-terminal amide. The transcript is expressed throughout development and is most abundant in heads. A custom FXPRLamide antibody revealed immunoreactive cells throughout the *Lygus* central nervous system consistent with typical neuropeptide expression. To assess potential functional roles of the peptides, a fluorescence-based Ca^2+^ influx assay using cultured insect cells stably expressing a moth PK receptor was performed. LyghePKa was unable to stimulate receptor activation, whereas LyghePKb triggered a robust response. The in vivo pheromonotropic activity of the two peptides was likewise assessed using three different moth species. Like the cell culture system, only the LyghePKb peptide was active. The study suggests evolutionary divergence of the PK gene in plant bugs and provides critical insights into likely biological functions in the western tarnished plant bug.

**Abstract:**

The pyrokinin (PK) family of insect neuropeptides, characterized by C termini consisting of either WFGPRLamide (i.e., PK1) or FXPRLamide (i.e., PK2), are encoded on the *capa* and *pk* genes. Although implicated in diverse biological functions, characterization of PKs in hemipteran pests has been largely limited to genomic, transcriptomic, and/or peptidomic datasets. The *Lygus hesperus* (western tarnished plant bug) PK transcript encodes a prepropeptide predicted to yield three PK2 FXPRLamide-like peptides with C-terminal sequences characterized by FQPRSamide (LyghePKa), FAPRLamide (LyghePKb), and a non-amidated YSPRF. The transcript is expressed throughout *L. hesperus* development with greatest abundance in adult heads. PRXamide-like immunoreactivity, which recognizes both *pk*- and *capa*-derived peptides, is localized to cells in the cerebral ganglia, gnathal ganglia/suboesophageal ganglion, thoracic ganglia, and abdominal ganglia. Immunoreactivity in the abdominal ganglia is largely consistent with *capa*-derived peptide expression, whereas the atypical fourth pair of immunoreactive cells may reflect *pk*-based expression. In vitro activation of a PK receptor heterologously expressed in cultured insect cells was only observed in response to LyghePKb, while no effects were observed with LyghePKa. Similarly, in vivo pheromonotropic effects were only observed following LyghePKb injections. Comparison of PK2 prepropeptides from multiple hemipterans suggests mirid-specific diversification of the *pk* gene.

## 1. Introduction

Insect neuropeptides regulate and/or modulate most physiological and behavioral processes [1,2,3,4]. Among the multitude of insect neuropeptides, the pyrokinin (PK) family is one of the most extensively studied. PKs are widely distributed throughout insects and are characterized by a core pentapeptide C-terminal motif (FXPRLamide) that is essential for biological functionality [5,6,7,8]. The founding member of the PK family, leucopyrokinin, was identified based on its ability to stimulate contractions in the cockroach hindgut [9]. In contrast, lepidopteran PKs, which were isolated soon after leucopyrokinin, were found to regulate sex pheromone biosynthesis [10,11], larval cuticular melanization [12,13], and the induction of embryonic diapause [14]. PKs are now recognized as highly conserved pleiotropic peptides that regulate a wide range of functionalities across the Insecta [15,16,17,18,19,20,21,22,23,24,25,26,27].

The PK family is composed of two peptide subfamilies that are differentiated by variations in the C-terminal motif and the type of receptor activated [5,6,7,8]. PK1 peptides, occasionally referred to as tryptoPKs or diapause hormone (DH)-like peptides, are characterized by a WFGPRLamide C-terminus. In contrast, PK2 peptides, such as the lepidopteran pheromone biosynthesis activating neuropeptides (PBANs), lack the Trp residue and have C terminal ends consisting of FXPRLamide (X = variable amino acid, but most frequently Gly, Ser, Thr, Val, or Ala) [5,6,7,8]. Although present in two different prepropeptides, the number and type of PK encoded on the respective genes are lineage dependent [5,6,7,8]. The *capa*/*periviscerokinin* (*pvk*) gene usually encodes a single PK1 along with multiple CAPA-PVKs; however, this is not always the case as the *capa*/*pvk* gene in fire ants has a PK2 peptide rather than a PK1 [28]. A PK1 peptide (i.e., DH) is likewise present in the lepidopteran *dh-pban* gene, which also encodes multiple PK2 peptides [29,30,31,32]. The homologous gene (i.e., *hugin*) in *Drosophila* [33], however, has lost the PK1 peptide. In non-lepidopterans, the peptides encoded on the *pk*/*dh-pban* gene can vary depending on the species with either both PK1 and PK2 peptides present or only PK2 peptides [6,34].

PKs impact diverse physiological functions across species including cuticle melanization [12,13], pheromone biosynthesis [10,11,25], pupariation [21,22], diapause [14,15,16,17], ecdysteroidogenesis [19], feeding behavior [27], and myotropic activity [9,20,26,33]. The role(s) PKs play in hemipterans, however, remains to be fully elucidated as functional characterization has largely been limited to in vitro assays using synthetic PKs from *Rhodnius prolixus* [35,36] and, more recently, PKs from *Halyomorpha halys* [37]. Despite the lack of functional data, hemipteran PKs are predicted in genomic and transcriptomic datasets [7,38,39,40,41,42,43,44] and processed peptides have been confirmed in multiple peptidomic datasets [42,44,45,46,47,48,49,50].

*Lygus hesperus* (western tarnished plant bug) is a destructive polyphagous agricultural hemipteran pest found throughout western North America [51,52,53]. While control strategies have largely been dependent on broad-spectrum insecticides, an over-reliance on insecticide applications could reduce field efficacy similar to that reported for the sister species *L. lineolaris* [54]. Ensuring the long-term utility of insecticides will require the adoption of alternative control approaches that complement existing technologies. One potential route is manipulation of peptidergic signaling systems. Given the range of physiological processes under its control, the PK system has been proposed as a candidate for targeted disruption [55,56,57,58]. While PK encoding transcripts homologous to the *capa* and *pk*/*dh-pban* genes are present in *L. hesperus* transcriptomic datasets [38,39], little is known about the expression and potential functionality of PKs in this species. Here, we utilized both RT-PCR and immunohistochemical imaging to examine the distribution of the Lygus PK2 encoding transcript (i.e., DH-PBAN like) and FXPRLamide peptides in *L. hesperus*. To assess the in vitro activity of the predicted *Lygus* PK2 peptides, we used an insect cell culture expression system heterologously expressing a lepidopteran PK2 receptor coupled to a Ca^2+^ influx reporter system. In addition, given the cross-species activity of PK2 peptides, we assessed the in vivo efficacy of the peptides using the well-defined lepidopteran assay that examines pheromone biosynthesis inducing (i.e., pheromonotropic) activity.

## 2. Materials and Methods

### 2.1. Insects

*L. hesperus* were obtained from a long-term laboratory colony (USDA-ARS Arid Land Agricultural Research Center, Maricopa, AZ, USA). The stock insects were given unrestricted access to a supply of bean pods (*Phaseolus vulgaris* L.) and an artificial diet mix [59] packaged in Parafilm M (Pechiney Plastic Packaging, Chicago, IL, USA) [60]. Both food sources were replenished as needed. Insects were reared at 27.0 ± 1.0 °C, 40–60% relative humidity (RH), under a L14:D10 (light:dark) photoperiod. Experimental insects were generated from eggs deposited in Parafilm M agarose packs with the resulting hatches maintained as above.

The *Mamestra brassicae* colony was established using adults collected from fields in different regions of Hungary. The stock colony was maintained in a rearing room at 25 ± 1 °C, 60% RH under a L16:D8 regime. Larvae were kept in large glass jars (18 × 14 cm), fed a semi-synthetic diet [61] smeared on the sides of the jar and covered with cloth. Prior to pupation, larvae were placed in a large bowl containing sterilized soil and pieces of the semi-synthetic diet. Pupae were collected from soil 8−10 days prior to eclosion and sexed. Females and males were separately maintained in 25 × 15 × 8 cm plastic containers on a layer of tissue paper. After eclosion, adults were placed in small glass jars (12 × 10 cm) covered with fine mesh and fed a 10% sterilized honey solution applied on cotton wool. The newly emerged individuals, considered to be day 0 (D0) adults, were culled every 24 h. Egg batches were cut from folded paper internally lining large mating jars that were covered with cloth.

A laboratory colony for the *E*-strain of *Ostrinia nubilalis* was established from two dozen larvae collected from maize stalks in Slovenia. Larvae burrowed into the semi-synthetic diet [61] provided at the bottom of large glass jars and were maintained under the same conditions as above except under a L18:D6 photoperiod. Larvae emerged from the diet to shelter and pupate in corrugated paper rolls. After a week, pupae were collected, sexed and kept in small glass jars humidified with wet cotton and covered with cloth. The age definition of emerging adults was as above. Egg batches were cut from plastic bags covering the inner walls of large mating jars.

*Spodoptera littoralis* were collected from a field in the Giza governorate (Egypt) and transferred to the Plant Protection Institute Hungary as pupae (with permission). All stages of *S. littoralis* were maintained in a separate room and reared under conditions similar to that described for *M. brassicae*. The number of experiments carried out was limited due to restrictions on colony size as *S. littoralis* is considered a potentially invasive species in Hungary.

### 2.2. Multiple Sequence Alignments and Phylogenetics

To examine phylogenetic relationships, multiple sequence alignments consisting of the putative *L. hesperus* PK prepropeptide and the prepropeptide sequences (Table S1) from 36 species representing five insect orders (Coleoptera, Diptera, Hemiptera, Hymenoptera, and Lepidoptera) were constructed using MUSCLE [62] with default settings implemented in Geneious Prime 2020.1.2 [63]. The evolutionary history was inferred with the maximum likelihood method using a JTT matrix-based model [64] implemented in MEGA X [65]. Initial tree(s) for the heuristic search were obtained automatically by applying Neighbor-Join and BioNJ algorithms to a matrix of pairwise distances estimated using the JTT model, and then selecting the topology with a superior log likelihood value. A discrete gamma distribution was used to model evolutionary rate differences among sites with 5 categories (+G, parameter = 2.4405). The rate variation model allowed for some sites to be evolutionarily invariable ([+I], 1.95% sites). This analysis involved 39 amino acid sequences. All positions with less than 95% site coverage were eliminated. Fewer than 5% alignment gaps, missing data, and ambiguous bases were allowed at any position (partial deletion option). There was a total of 77 positions in the final dataset. Prepropeptide sequences for the human and mouse neuromedin U, a vertebrate neuropeptide characterized by a C-terminal PRXamide that activates receptors in the same class as PKs [66], were used as outgroups. Phylogenetic analyses using minimum evolution and neighbor joining methods generated phylogenetic trees with similar topologies (data not shown).

To assess order-specific sequence conservation, a separate MUSCLE-based multiple sequence alignment was generated with a subset of hemipteran PK prepropeptides representing 12 species from six families. The alignment was generated with default settings as before but without the predicted signal peptide. Signal peptide prediction was performed using the Signal P5.0 server with eukarya settings [67]. Potential peptide cleavage sites were predicted using NeuroPred (http://stagbeetle.animal.uiuc.edu/cgi-bin/neuropred.py (accessed on 5 August 2021)) with model selection set to insect [68].

### 2.3. RT-PCR-Based Expression Profiling

TRI Reagent (ThermoFisher Scientific, Waltham, MA, USA) and RNeasy mini kit spin columns (Qiagen, Germantown, MD, USA) were used to isolate and purify total RNAs from two biological replicates of pooled eggs, nymphs (1st–5th instars), newly emerged adults (day 0), and sexually mature seven-day-old adults (day 7) as well as the heads, thoraces, and abdomens of mature adults. First-strand cDNAs were generated from 500 ng DNase I-treated total RNAs using Superscript III reverse transcriptase (ThermoFisher Scientific) and custom made random pentadecamers (IDT, San Diego, CA, USA). The full-length 396-nt *L. hesperus* PK prepropeptide transcript (*LyghePK*; KX584427) was amplified using Sapphire Amp Fast PCR Master Mix (Takara Bio USA Inc., Mountain View, CA, USA) in 20-μL volumes with 0.5 μL cDNA template and 0.2 μM of each primer (sense: 5′-ATGGTCAACCTGACAGCG; antisense: 5′-TTAATTCAATGTTATTCTGCGAAACC). As a positive indicator of cDNA quality, a 501-bp fragment of *L. hesperus* actin (GBHO01044314.1) was likewise amplified (sense: 5′-ATGTGCGACGAAGAAGTTG; antisense: 5′-GTAGATCGGGACGGTGTG). Thermocycler conditions consisted of 95 °C for 2 min followed by 37 cycles at 95 °C for 20 s, 56 °C for 20 s, 72 °C for 30 s, and a final extension at 72 °C for 5 min. The resulting products were separated on 1.5% agarose gels using a Tris/acetate/EDTA buffer system and visualized with SYBR Safe (ThermoFisher Scientific). A subset of the reactions was sub-cloned into pCR2.1-TOPO TA (ThermoFisher Scientific) and sequence validated (Arizona State University DNA Core Laboratory, Tempe, AZ, USA). Gel images were obtained using either an AlphaImager gel documentation system (ProteinSimple, San Jose, CA, USA) or an Azure 200 gel imaging workstation (Azure Biosystems, Dublin, CA, USA) and then processed in Adobe Photoshop v21.2.4 (Adobe Systems Inc., San Jose, CA, USA).

### 2.4. Immunohistochemistry

The distribution of PK-like immunoreactivity in the *L. hesperus* central nervous system (CNS) was observed using a whole-mount immunocytochemistry method described previously [69,70]. A polyclonal antiserum was generated from a synthetic peptide corresponding to the C-terminal 16 amino acids (DPEQIDSRTKYFSPRLamide) of *Helicoverpa zea* PBAN (HelzePBAN) [71]. CNS, including cerebral ganglia (CRG), gnathal ganglia (GNG), thoracic ganglia (TG), and abdominal ganglia (AG) were dissected from more than 15 adult *L. hesperus* across three different trials. The tissues were dissected in a cold phosphate-buffered saline (PBS), fixed in PBS/10% formalin for 1 h, and then incubated in PBS containing 2% Triton X-100 (PBS-T) overnight. Tissues were incubated for 6 h with the polyclonal PBAN antiserum (1:2000), followed by a secondary antibody (goat anti-rabbit IgG-peroxidase 1:2000; Sigma A9169; Mendota Heights, MN, USA), and a rabbit peroxidase anti-peroxidase soluble complex antibody (1:400; Sigma P1291) in PBS-T. Tissues were rinsed three times after each incubation with PBS-T. After the last incubation, the tissues were washed with PBS and then incubated in 50 mM Tris-HCl buffer (pH 7.6) for 10 min. Immunoreactivity visualization was achieved with a solution of 3,3′-diaminobenzidine and urea-H_2_O_2_ (Sigma D4168; tablets dissolved in 1 mL of deionized water). After satisfactory color development, the tissues were transferred to PBS and dehydrated by serial incubation in solutions of 40–100% glycerol. The resulting tissues were examined under an ECHO revolve microscope equipped with a digital camera (ECHO, San Diego, CA, USA). No staining was observed in the control tissue prepared using the same procedure but without the polyclonal antiserum. Respective designations of the morphological structures and their nomenclature are based on recommendations by the Insect Brain Name Working Group [72].

### 2.5. Insect Cell Culture-Based Characterization of Heterologously Expressed PK Receptor Activation

Although a *Lygus* PK2 receptor has yet to be identified and characterized, PK2 receptors, such as the lepidopteran PBAN receptor (PBANR), can exhibit cross-species activation by FXPRLamide peptides [73]. To examine the efficacy of synthetic LyghePKa and LyghePKb on PK2 receptor activation, polyclonal Sf9 insect cell lines stably expressing *Bombyx mori* PBANR [74] were generated. Briefly, the receptor open reading frame was cloned into the pIB/V5-His-TOPO TA insect expression vector (ThermoFisher Scientific) and transfected into adherent Sf9 cells (Allele Biotechnology) using Cellfectin II (ThermoFisher Scientific). At 48 h post-transfection, cells cultured in Grace’s insect media (Gibco/ThermoFisher Scientific) with 10% fetal bovine serum (FBS; Gibco/ThermoFisher Scientific) were selected with 100 μg/mL blasticidin (ThermoFisher Scientific) for three weeks and then maintained indefinitely in Grace’s supplemented with 10% FBS and 10 μg/mL blasticidin. On the day of the experiment, 3–4 × 10^5^ polyclonal cells were seeded into individual wells of a black-walled, clear bottom, tissue-culture treated 96-well microplate (Corning, Corning, NY, USA). Ligand-induced Ca^2+^ influx was assayed using a Fluo-4 direct calcium assay kit (ThermoFisher Scientific) as described previously [75]. Images were collected for 250 s (1 image every 8 s) with synthetic peptides (1 μM final concentration) added in a 20 μL volume after 40 s and the ionophore control, ionomycin (10 μM final concentration), added after 200 s. LyghePKa (LTFARESRNSASFQPRSamide) and LyghePKb (DEESQFTETSRSPPFAPRLamide) were custom synthesized at 95% purity (United Biosystems Inc., Herndon, VA, USA). HelzePBAN (LSDDMPATPADQEMYRQDPEQIDSRTKYFSPRLamide) was purchased commercially (Bachem Americas, Inc., Torrance, CA, USA). Lyophilized peptides were re-suspended in H_2_O:methanol (50:50 *v*/*v*) then diluted into IPL41 insect medium (Gibco/ThermoFisher Scientific) prior to each assay. Fluorescence imaging was conducted using an FSX-100 fluorescence microscope (Olympus America, Center Valley, PA, USA) with single cell measurements processed using the Fiji image processing package for Image J [76]. Fluorescence values were determined by subtracting both non-specific background fluorescence and the average fluorescence intensity over the first 40 s. Values were expressed relative to the maximal ionomycin-induced value and time-course plots generated using Prism9 (GraphPad Software, San Diego, CA, USA).

### 2.6. In Vivo Characterization of Pheromonotropic Activity

#### 2.6.1. Injection of Synthetic Neuropeptides and Preparation of Pheromone Gland Extracts

Given the established role of PK2 peptides (e.g., PBANs) in lepidopteran pheromone biosynthesis [77,78], we sought to assess the potential in vivo pheromonotropic activity of synthetic LyghePKa and PKb in three species of moths, *M. brassicae*, *O. nubilalis*, and *S. littoralis*. Although slightly different species-specific protocols were used to examine pheromone blend titers, the general scheme utilized decapitated females that were maintained in Petri dishes with moist filter paper until treatment. Different doses of the Lyghe-PK peptides, respective positive control peptides (5 pmol) or distilled water (DW) alone were injected (2 μL) abdominally using a 10-μL Hamilton microsyringe. All synthetic lyophilized peptides were re-suspended in H_2_O:methanol (50:50 *v*/*v*) on ice, and then further diluted with DW to obtain a series of doses (0.125–20 pmol) for testing. For reference, pheromone glands (PGs) from two-day-old (D2) non-decapitated calling and pheromone producing females were also extracted and measured. Each assay was replicated at least three times.

*M. brassicae* females were decapitated on the first day after emergence, in the second hour of scotophase. They were treated with peptides on the following day during the fifth-sixth hour of scotophase. *M. brassicae* pheromonotropin (Mambr-PT; SLAYVQKVFENVEFVPRLamide) was custom synthetized by the Department of Medical Chemistry, University of Szeged, Hungary [79] and used as a positive control. After 90 min, PGs were excised with the help of tweezers and fine scissors and extracted individually in 150 μL *n*-hexane (Merck, Darmstadt, Germany) for 8 min. The extracts were transferred to a conical glass insert within a 1.5 mL vial suitable for gas chromatography-mass spectroscopy (GC-MS) analysis. After addition of the internal standard *Z*13-18Ald (300 ng/3 µL) (Pherobank BV, Wijk bij Duurstede, The Netherlands) the extracts were concentrated using a thermo-block (at 65 °C) to a final volume of 20 µL. The vials were then immediately sealed with a Teflon cap and stored at −30 °C until analysis. Each sample contained the extract of one PG and a minimum of five replicates/dose were performed.

D2 *O. nubilalis* (*E* strain) virgin females were decapitated at the first h of scotophase and 27 h later were injected with varying amounts of the respective peptides. *O. nubilalis* PBAN (OstnuPBAN; LPEKVPVTPSDSHDEVYSFKPDMEEIISRHNYFSPRLamide) was custom synthesized by CASLO ApS (Technical University of Denmark, Lyngby, Denmark) [32] and served as a positive control. After 90 min, samples containing 3 PGs were extracted with 20 µL *n*-hexane for 8 min. The extracts were then transferred to conical vials with 5 ng/5 µL of *E*8,Z10-14Ac (Pherobank BV) added as an internal standard. The samples were placed into 1.5 mL GC-MS vials, sealed and stored as described above. Each sample contained the extract of three pooled PGs and a minimum of three replicates/dose were performed.

For *S. littoralis*, decapitation was carried out on D1 adults prior to scotophase and peptides were injected a day later in the first h of scotophase. After 90 min, PGs from 4–5 females were pooled and extracted for 1 h at room temperature in 50 µL of *n*-hexane with a resulting final volume ~15 µL. The extracts were transferred to conical vials, and 500 ng/5 µL of 13:OAc (Sigma, Darmstadt, Germany) was added as an internal standard; samples were handled as above until analysis. Each sample contained the extract of 3–4 pooled PGs and a minimum of three replicates/dose was performed. The data were adjusted to reflect a single PG.

#### 2.6.2. Gas Chromatography-Mass Spectrometry Analysis

Measurements were carried out on a gas chromatograph-mass selective detector (Hewlett Packard GC 6890, HP MSD 5973) equipped with an automatic injector unit. The injection volume was 1 µL in splitless mode with helium (6.0) as a carrier gas at a flow rate of 1 mL/min. For the *M. brassicae* and *O. nubilalis* samples, a RESTEC (Rxi-5SI) column (0.25 mm internal diameter × 30 m and 0.25 µm film thickness) was used. The GC separation conditions were as described in Moustafa et al. [80] for *M. brassicae* and Fodor et al. [32] for *O. nubilalis*. The selected ion monitoring (SIM) method was used, and calculations were performed with MSD ChemStation ver. D.01.02.16. For *S. littoralis* samples, the same GC-MS unit was used, but with an Agilent J&W VF WAXms (60 m × 0.25 mm × 0.25 µm) polar capillary column. Running conditions were as described by Moustafa et al. [81]. Authentic standards were initially injected in scan mode. The SIM method was used for both quantitative mass spectrometric detection and to confirm compound identity by utilizing the NIST 17 mass spectral database. For quantitative evaluation, Mass Hunter Workstation Quantitative Analysis B.09.00 was used.

## 3. Results

### 3.1. L. hesperus PK Prepropeptide Transcript

**Figure 1:**
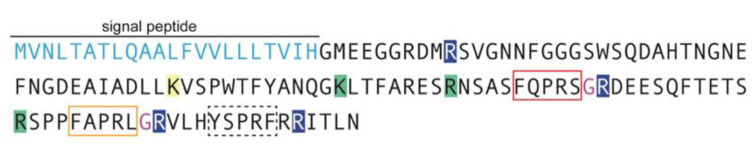
*L. hesperus* pyrokinin prepropeptide sequence. The predicted signal peptide is indicated in blue font. Carboxyl-terminal amidation signals are shown in magenta. Predicted cleavage sites based on NeuroPred probability outputs are highlighted in dark blue (>50% probability), green (1–10% probability), and yellow (<1% probability). An atypical FXPRLa sequence is indicated by the red box, a characteristic FXPRLa-like sequence by the orange box, and an FXPRL-like sequence that lacks the C-terminal amidation by the dashed box.

A *L. hesperus* PK (*LyghePK*) encoding transcript was previously identified in a whole-body transcriptomic dataset (GBHO01031681) and its sequence confirmed by cDNA cloning (KX584427) [38]. The transcript was also identified (GDHC01017048) in a more recent survey of the *L. hesperus* peptidome [39]. The 396-nt *LyghePK* transcript encodes a 131 amino acid prepropeptide with a predicted 23 amino acid N-terminal signal peptide and multiple endoproteolytic cleavage sites that, if processed, would yield peptides with three different PRXamide C terminal ends (Figure 1). Cleavage at Lys77 would yield a 17-amino acid peptide (LTFARESRNSASFQPRSamide; LyghePKa) with an atypical pentapeptide motif; cleavage could also occur at Arg85 to yield a truncated 9-amino acid form of the peptide. Cleavage at Arg96 would generate a 19-amino acid peptide (DEESQFTETSRSPPFAPRL; LyghePKb) with the characteristic FXPRLamide C-terminus. Similar to LyghePKa, a potential internal cleavage site (Arg107) could generate a smaller, 8-amino acid form of the peptide. The last PRXamide peptide (VLHYSPRFR; LyghePK-like) would be generated by cleavage of Arg117 and Arg127; however, the resulting peptide would have a non-amidated C-terminus and differ from the characteristic pentapeptide at positions 1 (Tyr121) and 5 (Phe125). All of the predicted peptides fit the PK2 designation (i.e., they lack a Trp residue immediately upstream of the core pentapeptide) and no PK1 peptides are encoded on the transcript.

**Figure 2:**
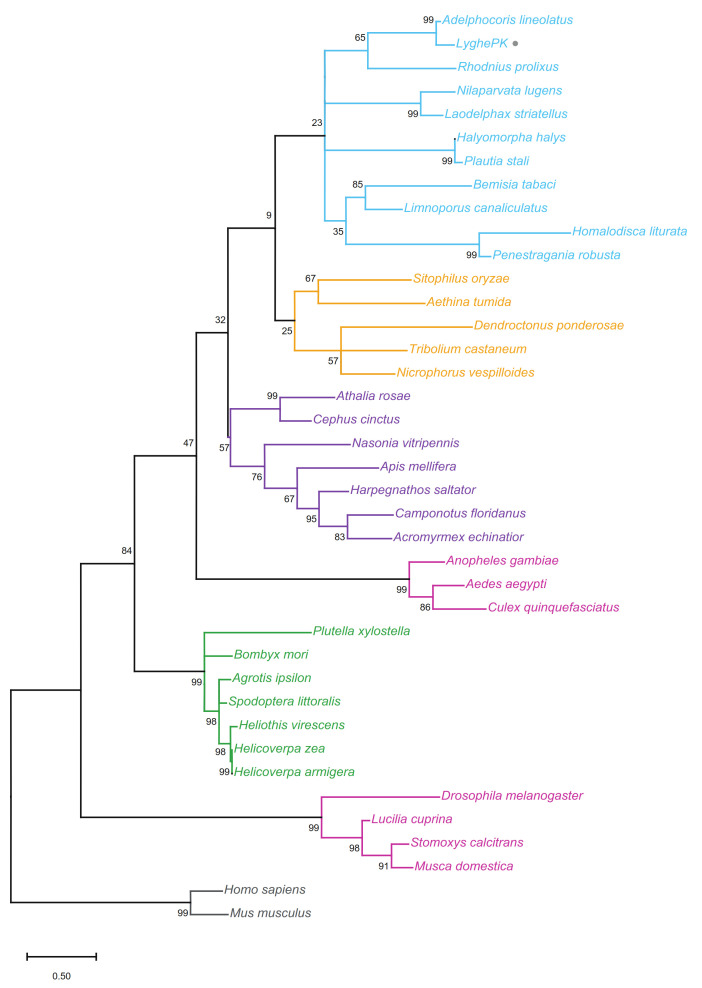
Phylogenetic relationship of the putative *Lygus hesperus* pyrokinin prepropeptide with sequences from multiple species across five insect orders. The highest log likelihood (−4698.53) tree is shown with the percentage of trees that clustered together over 1000 iterations indicated at the branch nodes. The tree is drawn to scale, with branch lengths measured in the number of substitutions per site. LyghePK is marked by a grey circle. Order specific clades have been colored as: Coleoptera—orange; Diptera—magenta; Hemiptera—blue; Hymenoptera—purple; and Lepidoptera—green. The tree was rooted to the human and mouse neuromedin U (the vertebrate PK homolog) sequences (gray). Accession numbers for the proteins used are listed in Table S1.

The evolutionary relationship of the LyghePK prepropeptide with known or predicted PK prepropeptides from 36 species, representing five insect orders, was inferred through phylogenetic analyses. A maximum likelihood-based tree with bootstrap support shows clear delineation at the order level, with the LyghePK sequence sorting to a branch in the hemipteran clade (Figure 2). Less robust bootstrap support for the more ancestral order-specific nodes likely reflects the higher rate of divergence observed in the prepropeptide sequences. The observed bifurcation of dipterans into two suborder specific (Brachycera and Nematocera) clades is consistent with previous findings [82]. 

**Figure 3:**
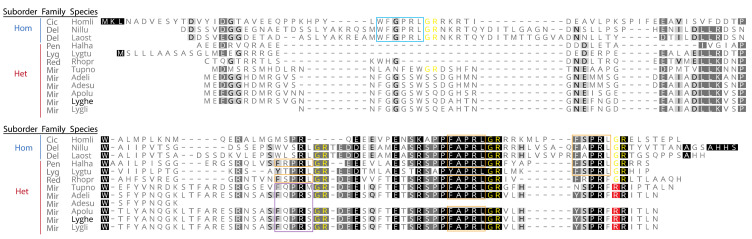
Alignment of hemipteran PK-like prepropeptides. The *Lygus hesperus* PK (LyghePK) sequence is indicated in black font. PK species abbreviations and accession numbers are: Homli (*Homalodisca liturata*; GECU01033249.1); Nillu (*Nilaparvata lugens*, BAO00974.1); Laost (*Laodelphax striatellus*, RZF43903.1); Halha (*Halyomorpha halys*, AYP97818.1); Lygtu (*Lygaeus turcicus*, GCYB01067468.1); Rhopr (*Rhodnius prolixus*, ADA83379.1); Tupno (*Tupiocoris notatus*, GFBA01045781.1); Adeli (*Adelphocoris lineolatus*, GGBQ01031033.1); Adesu (*Adelphocoris suturalis*, GGBU01027748.1); Apolu (*Apolygus lucorum*, KAF6216512.1); Lygli (*Lygus lineolaris*, GCXM01012899.1). Family abbreviations are: Cic, Cicadellidae; Del, Delphacidae; Pen, Pentatomidae; Lyg, Lygaeidae; Mir, Miridae. Suborder abbreviations are: Hom, Homoptera; Het, Heteroptera. Potential processing sites that result in C-terminal amidation are shown in yellow font. PK1 sequences are boxed in blue, FXPRL sequences characteristic of PK2s are boxed in orange, a mirid specific FXPRL-like motif (FQPRS/M) is boxed in purple, and mirid specific losses of C-terminal amidation sites are highlighted in red. The alignment was performed with predicted signal peptides excluded.

A more focused analysis of the LyghePK prepropeptide sequence in relation to other hemipteran *pk*/*dh-pban* derived PK prepropeptides revealed significant divergence at the suborder level, with homopterans encoding a PK1 peptide that has been lost in heteropterans (Figure 3). Family specific divergences were also observed with the first PK-like sequence in mirid prepropeptides characterized by an FQPRS/Mamide and the last PK-like sequence by a non-amidated Y/NSPRF pentapeptide.

### 3.2. RT-PCR Expression Profiling

**Figure 4:**
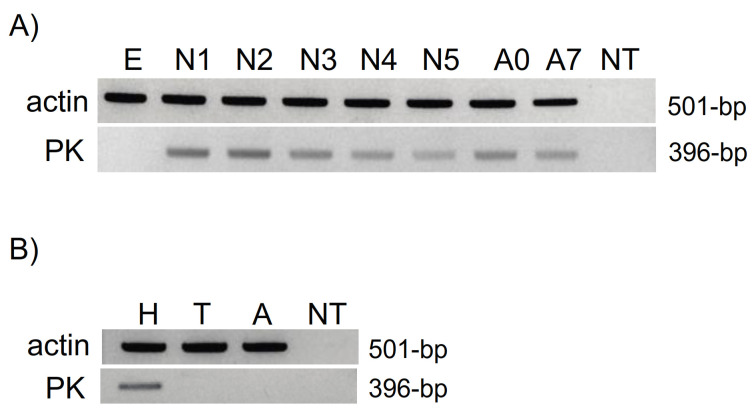
Endpoint RT-PCR expression profile of the *LyghePK* transcript. (**A**) Expression across *L. hesperus* development. Abbreviations: egg, E; 1st–5th instar nymphs are N1-N5, and adults aged 0 and 7 days post-eclosion are A0 and A7. (**B**) Expression in mixed sex adult body segments. Abbreviations—H, head; T, thorax; A, abdomen. NT denotes reactions lacking a cDNA template.

To provide insights into potential functional roles, we examined the expression of *LyghePK* transcripts in eggs, nymphs, and adults. With the exception of eggs, *LyghePK* was expressed to varying degrees from all stages (Figure 4A). We also assessed *LyghePK* transcript localization in head, thorax, and abdominal segments of seven-day-old adults. Clear amplification was observed in head cDNAs (Figure 4B), which is consistent with previous studies indicating subesophageal ganglion (SEG) localization of PK2 encoding *dh-pban*-like transcripts and peptides [17,33,41,45,47,48]. Sequence validated amplicons were also generated from both thorax and abdomen cDNAs (Figure S1A), albeit inconsistently across biological groups, suggesting low abundance and/or conditional expression of *LyghePK* transcripts. The products were not derived from genomic DNA as total RNAs that were not reverse transcribed did not yield a product (Figure S1B).

### 3.3. Localization of FXPRLamide-like Immunoreactivity in the CNS

**Figure 5:**
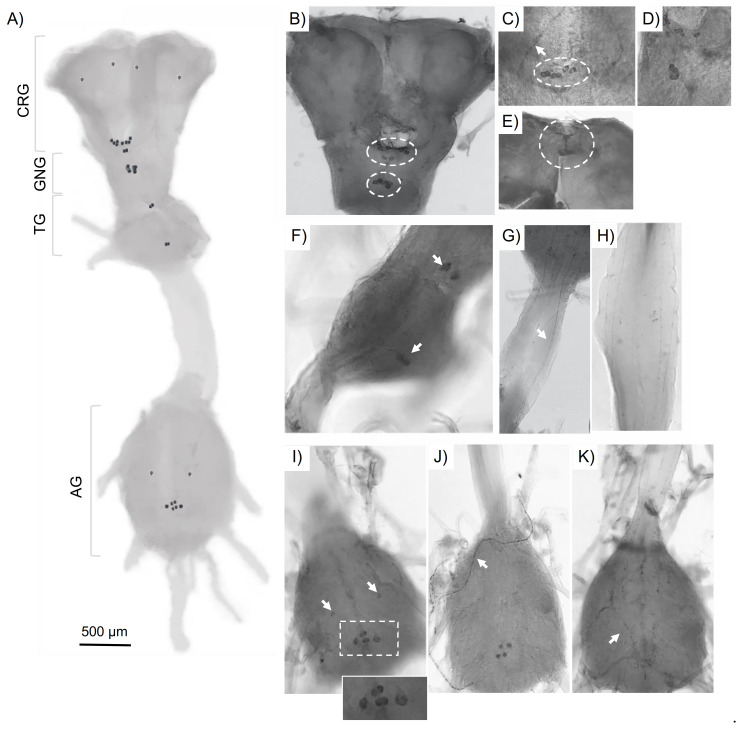
Localization of FXPRL-like immunoreactivity in the *Lygus hesperus* central nervous system (CNS). (**A**) Schematic diagram of the CNS which includes the cerebral ganglia (CRG), gnathal ganglia (GNG), thoracic ganglia (TG), and abdominal ganglia (AG) with the corresponding FXPRLamide-like immunoreactive neurosecretory cells indicated by black dots. (**B**) Location of somata clusters in the CRG (upper circle) and GNG (lower circle). (**C**) Magnified image of immunoreactive varicosities (arrow) and neurites from axons in the CRG that project through the median bundle, which has a densely stained region of cells (circle). (**D**) Magnified image of densely stained region around the esophagus zone. (**E**) Immunoreactivity in the retrocerebral complex. (**F**) Two pairs of neurosecretory cells in the TG (arrows). (**G**) The ventral view of at least three pairs of dendrites (arrow) that project into the AG from the TG or GNG. (**H**) The dorsolateral view of the dendrites. (**I**) Three pairs of somata along the AG median line (boxed and inset) that likely correspond to *capa*-derived peptides and a pair of weaker immunoreactive cells more posterior (arrows). (**J**) Neurohemal organ (arrow) of the ventral ganglia. (**K**) Varicosities (arrow) present throughout the ventral ganglia.

To determine the specific CNS localization of the *Lygus* PK2 peptides, immunohistochemical imaging was done using a custom antiserum that recognizes the characteristic FXPRLamide C-terminus, but which is unable to differentiate between peptides encoded by the *Lygus* CAPA homolog (MT210027) from LyghePKa and/or PKb. The morphological structures and names of the *Lygus* CNS (Figure 5), including cerebral ganglia (CRG), gnathal ganglia (GNG, formerly referred to as the SEG), thoracic ganglia (TG), and abdominal ganglia (AG) are based on the systematic nomenclature suggested by the Insect Brain Name Working Group [72]. Two clusters of immunoreactive cells were found in the ventral CRG, with the dorsolateral part of each protocerebrum containing two pairs of FXPRLamide neurosecretory cells (Figure 5A). The projection of the neurites within the cerebrum was not determined. An additional set of at least five pairs of strongly immunoreactive neurosecretory cells (upper circle in Figure 5B) in the *Lygus* CRG localized to the fused deutocerebrum (DE) and tritocerebrum (TR). Axonal projections of the DE and TR in the CRG extend through the median bundle of the CRG, where they form a densely stained region of varicosities (Figure 5C,D). Clear immunoreactivity was also seen in regions corresponding to the retrocerebral complex consisting of the *corpora cardiaca* and *corpora allata* (Figure 5E), which is consistent with previous reports on CNS localization of PK2 peptides [28,41,45,47]. At least three pairs of immunoreactive neurosecretory cells were present in the mandibular, maxillary, and labial regions of the GNG (Figure 5A,B lower circle) with two more pairs of immunoreactive neurons in the fused TG (Figure 5A,F). Further down the ventral nerve cord, neurites that appear to connect with the AG were stained along their lateral lines (Figure 5G,H). In the AG (Figure 5I,J), three pairs of cells along the median line (Figure 5I, inset) were immunoreactive, which likely corresponds to *capa*-derived peptides (i.e., PVK and PK1) in abdominal segmental nerves similar to that seen in a number of insect species [28,46,47,49]. Unexpectedly, a pair of weak immunoreactive cells was also observed in the anterior portion of the AG (Figure 5I, see arrows). Whether the peptides processed in these cells are also *capa*-derived remains to be determined. However, the presence of PK2 encoding transcripts in abdominal segments (Figure S1) would be consistent with PK2 cross-reactivity. Projections from the AG cells to neurohemal sites for peptide release into the hemolymph were also observed (Figure 5J). In addition, numerous varicosities were found throughout the ventral ganglia (Figure 5K).

### 3.4. Heterologous PK2 Receptor Activation

**Figure 6:**
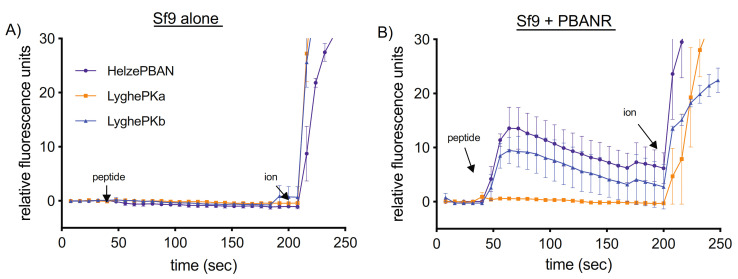
In vitro activation of a heterologously expressed PK2 receptor. Extracellular Ca^2+^-based intracellular fluorescence signals were quantified over time in (**A**) Sf9 cells alone and in (**B**) Sf9 cells stably expressing the *B. mori* PBAN receptor (PBANR). Peptides (1 μM) were added 40 s into the scan period with the positive control, 10 μM ionomycin (ion), added at 200 s. Each point is the mean value ± SEM of 25 individual cells in triplicate.

To assess the potential receptor activation capabilities of LyghePKa and LyghePKb, Ca^2+^ influx assays were performed using cultured insect Sf9 cells stably expressing a lepidopteran PK2 receptor, *B. mori* PBANR [83]. Although 8–9 amino acid versions of the respective Lygus PK2 peptides may be generated in vivo (Figure 1), we elected to utilize longer versions of the peptides (LyghePKa—LTFARESRNSASFQPRSamide; LyghePKb—DEESQFTETSRSPPFAPRLamide) to more closely align with our control peptide, Helze-PBAN (LSDDMPATPADQEMYRQDPEQIDSRTKYFSPRLamide). Wildtype, non-transformed Sf9 cells failed to respond to any of the synthetic peptides tested (Figure 6A). In contrast, Ca^2+^ responses were detected in Sf9 cells stably expressing *B. mori* PBANR following addition of LyghePKb and HelzePBAN, but not LyghePKa (Figure 6B).

### 3.5. In Vivo Pheromonotropic Activity

**Figure 7:**
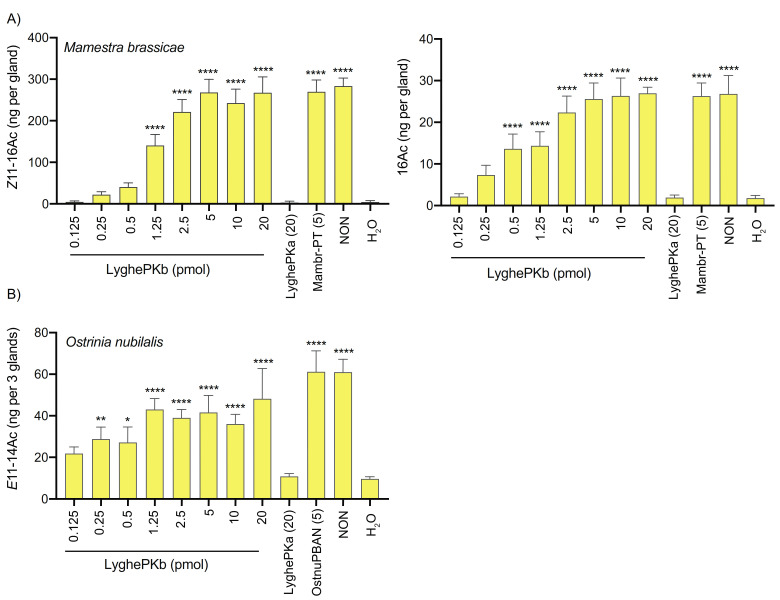
In vivo pheromonotropic activity of *Lygus* PK2-like peptides. Varying amounts of synthetic LyghePKa and PKb were injected into decapitated *Mamestra brassicae* (**A**) or *Ostrinia nubialis* (**B**) adult females. Sex pheromone production assays were conducted 90 min post-injection, with results shown for the components Z11-16Ac and 16Ac in *M. brassicae*, (**A**) and E11-14Ac in *O. nubilalis* (**B**). Positive controls consisted of 5 pmol synthetic *M. brassicae* pheromonotropin (Mambr-PT) or 5 pmol synthetic *O. nubilalis* PBAN (OstnuPBAN). Non-decapitated controls (pheromone emitting females actively calling) are indicated as “NON” and water-injected, decapitated females are indicated as “H_2_O”. Bars represent the mean ± SD of at least 3 replications. Statistical differences from water-injected females determined via ANOVA with Dunnett’s correction for multiple comparisons (* *p* < 0.05; ** *p* < 0.01; **** *p* < 0.0001).

Given the well-established pheromonotropic role of PK2 peptides in lepidopterans [77,78], we next sought to determine if the *Lygus* peptides could stimulate pheromone biosynthesis in different moth species by injecting varying amounts of synthetic LyghePKa and LyghePKb into decapitated females. Depending on geographical region, the *M. brassicae* sex pheromone blend consists of 2–3 components with the two predominant compounds, (*Z*)-11-hexadecenyl acetate (*Z*11-16:Ac) and hexadecyl acetate (16:Ac), present in a 93:7 ratio [79]. LyghePKb exhibited dose-dependent effects on *M. brassicae* pheromone production (Figure 7A), with maximal values comparable to those induced by 5 pmol Mambr-PT, a well-documented synthetic octadecapeptide with pheromonotropic activity [84]. In contrast, the pheromonotropic effect of LyghePKa was indistinguishable from water alone (Figure 7A), with no pheromonotropic activity observed even after 50 pmol injections (data not shown).

Although the *E*-strain of *O. nubilalis* utilizes a two component sex pheromone blend of (*E*)-11- and (*Z*)-11-tetradecenyl acetate (*Z*11-14:Ac and *E*11-14:Ac), our assays focused on the *E*11-14:Ac major component because *E*-strain moths produce a 97:3 ratio of *E*11-14:Ac and *Z*11-14:Ac [85]. Injections with synthetic LyghePKb stimulated pheromone production, albeit without the clear dose-dependence observed in *M. brassicae*, and its pheromonotropic activity was comparable to that of the synthetic OstnuPBAN positive control (Figure 7B). LyghePKa effects were again indistinguishable from those of water alone (Figure 7B).

At least five C14 acetates have been identified in pheromone gland extracts of *S. littoralis* [81,86,87]. We consequently examined the effects of the synthetic PKs on levels of all five compounds in the PGs (Table 1). While (*Z*,*E*)-9,12–14:Ac levels were not affected by PK administration, all others exhibited significant increases following injections of LyghePKb at 5 and 20 pmol, but not 1.25 pmol. As with the other species assayed, the effects of LyghePKa injection were negligible on pheromone levels.

## 4. Discussion

PKs, one of the most extensively studied neuropeptide families, mediate numerous functions across insects [6,7]. The PK encoding genes in insects, *capa/pvk* and *pk/dh-pban*, appear to be evolutionarily derived from an ancestral hexapod gene that encoded three peptide subfamilies, CAPA, PK1, and PK2 [34]. The *capa/pvk* gene typically encodes two CAPA-PVKs followed by a single PK1 [7], but in some species can also encode a C-terminal PK2-like peptide that is selectively processed [41,88], or as in fire ants, encode a PK2 peptide rather than the PK1 peptide [28]. In contrast, the *pk/dh-pban* gene exhibits greater sequence variation, with the number and type of PKs encoded per gene being lineage-dependent [7]. Among *pk/dh-pban* genes identified to date, the predominant configuration of the transcribed products has the PK1 peptide situated near the signal peptide, with PK2 or PK2-like peptides further downstream. The *LyghePK* transcript, however, only encodes PK2-like peptides. Loss of the PK1 peptide from the *pk/dh-pban* gene likely occurred in the Hemiptera following divergence of the homopteran and heteropteran lineages (Figure 3 and [41]). Similar loss appears to have also independently occurred multiple times [34] including in the dipteran suborder Brachycera (e.g., *Drosophila melanogaster* [33]; *D. suzukii*, [89]) and the sandfly *Phlebotomus papatasi* [82]. This latter loss, however, does not permeate the Nematocera suborder as the PK1 peptide has been retained in mosquitoes [90]. In *L. hesperus*, a PK1 peptide is encoded by the *capa/pvk* transcript (MT21002), which consists of a 531-nt open reading frame that has the PK1 peptide downstream of two CAPA-PVK-like peptides [39]. The presence of two PK1 peptide generating genes that are differentially expressed [28,41] suggests the respective peptides may have different functional roles. For species without the *pk/dh-pban* derived PK1, the lost functionality may be provided by a different peptide. Alternatively, given cell-dependent differential processing of prepropeptides [88,91,92], it is possible that the ancestral *pk/dh-pban* derived PK1 was not post-translationally processed into a bioactive peptide and was subsequently lost as a non-functional element of the *pk/dh-pban* gene in heteropterans.

The biologically active forms of neuropeptides are generated by proteolytic processing of the peptide precursors. Although this processing typically occurs at sites demarcated by mono- or dibasic amino acids [93], the predicted usage of potential sites from sequence information can be complicated by differences in processing between phyla/species [91,94] as well as tissue/cell-specific variation [47,49,95]. The NeuroPred prediction algorithm identified Arg32, Arg96, Arg117, and Arg127 as high probability (>50%) cleavage sites with the latter three generating the C-terminal ends of the *Lygus* PK-like peptides. Additional cleavage sites (Lys77, Arg85, and Arg107) had significantly lower probability. Cleavage at Lys77 and Arg96 would generate the 17-amino acid peptide (LTFARESRNSASFQPRSamide) that we have termed LyghePKa. The Arg85 site is 8-amino acids downstream of Lys77 and if cleaved would be expected to yield the 9-amino acid peptide NSASFQPRSamide. This peptide is analogous to the NTVNFRPRLamide peptide detected in *C. lectularius* [47] and the pQLVSFRPRLamide (i.e., PK-2) detected in multiple species of stink bug [45]. In all these peptides, the N-terminal cleavage site is an Arg residue four residues upstream of the characteristic PK2 pentapeptide sequence. Conservation of this Arg in other mirids (Figure 3) could indicate that it is a true in vivo cleavage site. Processing of Arg96 and Arg117 would yield the 19-amino acid peptide (DEESQFTETSRSPPFAPRLamide) that we have termed LyghePKb. Cleavage of Arg107 would yield an 8-amino acid peptide (SPPFAPRLamide) that has been detected via mass spectrometry methods in multiple hemipteran species [45,46,47,48,49]. Extended forms of the peptide (e.g., EEDIIFTETSRSPPFAPRLamide in *C. lectularius*) were also detected and the longer peptide was the only one detected in *Diaphorina citri* [50], suggesting incomplete cleavage of the Arg107 site may be common or that its utilization may vary in a cell/tissue and species-specific manner. Direct detection of the various processed peptides in mass spectra of the *Lygus* retrocerebral complex is needed to confirm which cleavage sites, if any, are utilized in vivo.

PK2 encoding prepropeptides have been localized to three neuronal clusters in insect brain ganglia with release sites centered around the retrocerebral complex [5,33,41]. The PK immunocytochemical profile in *L. hesperus* is largely consistent with this pattern (Figure 5). The immunoreactivity in the AG, however, is less straight forward. The antibody utilized in this study will recognize all PRXamide peptides including those generated by the *capa*/*pvk* gene, which in *Lygus* is predicted to yield three PRXamides—two CAPA-PVK-like peptides (DTSGLIPFPRVamide and QESGLIPFPRSamide) and one PK1 (NGAGSGGSLWFGPRLamide) [39]. Furthermore, only *capa*-derived peptides have been identified in mass spectra datasets of abdominal nerve preparations across insect species [28,46,47,49]. Consequently, the staining profile of the three cells along the AG median line likely reflects this cross-reactivity. The other pair of cells in the posterior portion of the AG (Figure 5I, arrows) may also correspond to *capa/pvk* peptides; however, validated amplification of the *LyghePK* transcript from the abdominal segment (Figure S1), albeit sporadic, could indicate that the observed immunoreactivity may be attributable to the *Lygus* PK2 peptides. Although abdominal expression of the *pk/dh-pban* gene has been likewise reported for *H. halys* [41], it has been largely undetectable in species across multiple orders [82,90,96]. Additional studies with other hemipterans (both heteropteran and homopteran) will be needed to determine if the expression of PK2 encoding transcripts in the abdominal segment is limited to *Lygus* and *H. halys* or if it is more widespread. The expression of the *LyghePK* transcript throughout nymphal and adult development is consistent with that observed previously in other insect species [28,82,89,90] and suggests that the LyghePK peptides may play a role in mediating more generalized, multi-physiological functions.

Although functional characterization of hemipteran PK2 peptides has been limited to in vitro receptor activation assays [35,36], cross-species reactivity of the core FXPRLamide C-terminal pentapeptide has identified essential physiochemical properties of the peptide. The critical importance of the C-terminal amide has been demonstrated both in vivo [11,97,98] and in vitro via receptor activation assays [99] and is the reason why the LyghePK2-like peptide (VLHYSPRF), which is predicted to have a non-modified C- terminal free acid, was not assayed. Among the lepidopteran PK2 peptides that have been assayed, the amino acid at position 2 typically consists of a smaller amino acid characterized by either a hydrophobic or uncharged polar side chain (e.g., Gly, Val, Ser, Thr) with pheromonotropic activity reported to be highest with Thr compared to Val, Ser, or Gly [100]. In non-lepidopteran species, the presence of an FAPRLamide pentapeptide (i.e., LyghePKb) is well-conserved [41,45,46,47,48,50,82]. Furthermore, Ala substitution of position 2 in *H. zea* PBAN had negligible effects on in vitro receptor activation [101], indicating that the substitution has little impact on receptor interactions. The absence of detectable activity in either the receptor activation (Figure 6) or pheromonotropic (Figure 7) assays by LyghePKa (FQPRSamide) could indicate that the Gln at position 2, which introduces a larger uncharged side chain, impedes peptide binding. The *Drosophila* PK2 pentapeptide (FKPRLamide), however, has a Lys substitution that does not impede receptor activation [89]. Alternatively, the lack of activity seen with LyghePKa may be attributable to the Ser substitution of the near invariant Leu. Ala substitution of this residue in *H. zea* PBAN led to significantly reduced in vitro receptor activation [101] and PVK peptides characterized by a PRVamide C terminus do not activate the *Drosophila* PK2 receptor [66]. The absence of activity observed with LyghePKa might also be linked to a potentially extended N terminus in the synthetic peptide (LTFARESRNSASFQPRSamide) that may sterically disrupt interactions between the receptor binding pocket and the pentapeptide C terminus. Because proteolytic processing of the LyghePK prepropeptide has yet to be empirically determined, we designed the synthetic peptide based on potential processing from Lys77. Utilization of Arg85 as the cleavage site would generate a shorter LyghePKa peptide that may not exhibit the steric hindrance problems of the longer peptide. The bioactivity of lepidopteran PBANs, which are typically 33 amino acids in length, however, suggest that this putative steric effect is likely negligible. Regardless, the presence of atypical FXPRLamide pentapeptides in the LyghePK prepropeptide might indicate that the cognate receptor, which has yet to be functionally characterized, may have a more accommodating binding pocket than the lepidopteran PK2 receptor (PBANR) assessed in this study.

PK2 and PK2-like peptides have been implicated in a number of physiological responses, including pheromonogenesis [10,11], melanization [12,13], embryonic diapause [14], pupal diapause [15,16,17], seasonal polyphenism [18], ecdysteroidogenesis [19], myostimulation [20], puparium formation [21,22], sex pheromone synthesis in male heliothine moths [23,24], ant trail pheromone biosynthesis [25], and mosquito hindgut motility [26]. The in vivo role of the peptides in hemipterans, however, remains to be fully elucidated. Elevated expression of PK2 receptors in *H. halys* Malpighian tubules and reproductive tissues [37] could indicate potential roles for PK2 in osmotic balance and/or reproductive behaviors. In aphids, biostable PK2 pentapeptide (FGPRLamide and FTPRLamide) analogs have been reported to have antifeedant/aphicidal effects following injection, topical application, and feeding [58,102,103]. The physiological roles of these peptide analogs, however, have yet to be determined in other hemipterans. While the expression profile reported here is consistent with a myostimulatory/inhibitory role for the LyghePKs, biochemical verification of the prepropeptide processing pattern and/or physiological determination of its in vivo activity are needed.

## Figures and Tables

**Table 1:** Pheromone biosynthesis in adult *Spodoptera littoralis* females.

Treatment	*Z-9-14:Ac*	*E-11-14:Ac*	*Z-11-14:Ac*	*(Z,E)-9,12–14:Ac*	*(Z,E)-9,11–14:Ac*
control	**1.55 ± 0.18**	1.11 ± 0.49	0.54 ± 0.20	3.09 ± 2.08	**3.25 ± 0.67**
Mambr-PT (5 pmol)	**5.14 ± 0.55**	**3.13 ± 0.58**	**1.59 ± 0.33**	4.09 ± 0.4	**9.10 ± 0.46**
water-injected	0.25 ± 0.10	0.22 ± 0.06	0.02 ± 0.02	3.19 ± 1.16	0.41 ± 0.14
LyghePKb (20 pmol)	**2.4 ± 0.75**	**1.90 ± 0.70**	**0.98 ± 0.39**	5.30 ± 1.77	**3.97 ± 0.3**
LyghePKb (5 pmol)	**1.92 ± 0.52**	**1.83 ± 0.1**	**1.11 ± 0.55**	3.77 ± 2.99	**4.56 ± 1.18**
LyghePKb (1.25 pmol)	0.49 ± 0.25	0.42 ± 0.16	0.70 ± 0.49	3.75 ± 0.65	1.83 ± 0.32

Mean pheromone blend component titers ng/female ± SE (CV%, SE/Mean, *n* = 4–5 pheromone glands/sample in three replicates) of 2-day-old *S. littoralis* females (2–3 h into scotophase). Values shown in bold are significantly different from water-injected controls via ANOVA with Dunnett’s correction for multiple comparisons (*p* < 0.05).

## Supplementary Materials

The following are available online at https://www.mdpi.com/article/10.3390/insects12100914/s1, Figure S1: (A) Inconsistent *LyghePK* transcript amplification across multiple biological replicates; (B) *LyghePK* amplification is dependent on reverse transcriptase (RT) positive templates. Table S1: Accession numbers of proteins used in phylogenetic analyses.
