# Supplementary material for: Molecular and Functional Characterization of Pyrokinin-Like Peptides in the Western Tarnished Plant Bug Lygus hesperus (Hemiptera: Miridae)
Source: Insects. 2021 Oct 6;12(10):914. doi: 10.3390/insects12100914 (PMC8541414; doi:10.3390/insects12100914)
Supplement: Supplementary file 1 [file insects-12-00914-s001.zip › [Insects] insects-1385913-Supplementary materials/Fig S1 R3.pdf]

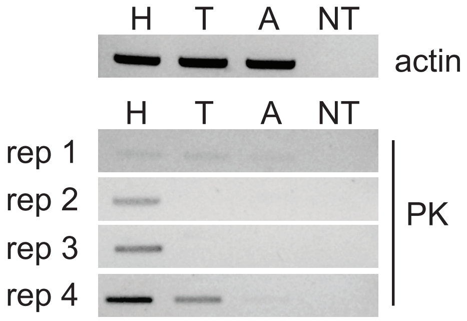

Figure S1A. Inconsistent *LyghePK* transcript amplification across multiple biological replicates. RT-PCR was performed using mixed sex adult body segment cDNAs prepared from four separate biological replicates (reps 1-4). Abbreviations - H, head; T, thorax; A, abdomen. NT denotes reactions lacking a cDNA template. Representatiave products from each segment were sub-cloned and sequenced.

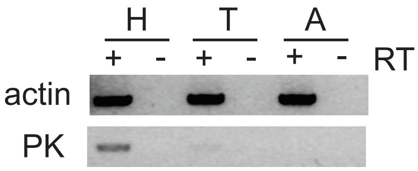

Figure S1B. *LyghePK* amplification isdependent on reverse transcriptase (RT) positive templates. Total RNAs that were not reverse transcribed did not yield a product, indicating that DNase treatment of total RNAs prior to cDNA synthesis sufficiently removed any gDNA contamination. Abbreviations - H, head; T, thorax; A, abdomen.
